# Anti-HMGCR myopathy: a first case report from North Africa and literature insights

**DOI:** 10.3389/fimmu.2025.1590913

**Published:** 2025-05-12

**Authors:** Houssem Abida, Imen Zamali, Imène Rachdi, Zakaria Saied, Ahlem Ben Hmid, Samar Samoud, Yousr Galai, Fatma Daoud, Fatma Boussema, Samia Ben Sassi, Zohra Aydi, Mélika Ben Ahmed

**Affiliations:** ^1^ Faculty of Medicine de Tunis, University of Tunis El Manar, Tunis, Tunisia; ^2^ Laboratory of Clinical Immunology, Institut Pasteur de Tunis, Tunis, Tunisia; ^3^ Laboratory of Transmission, Control and Immunobiology of Infection, Institut Pasteur de Tunis, Tunis, Tunisia; ^4^ Internal medicine department, Habib Thameur Hospital, Tunis, Tunisia; ^5^ Neurology department, National Institute of Neurology Mongi Ben Hamida, Tunis, Tunisia

**Keywords:** case report, autoimmunity, anti-HMGCR, inflammatory myopathy, therapeutics

## Abstract

Anti-3-hydroxy-3-methylglutaryl-coenzyme A reductase (anti-HMGCR) myopathy is a rare idiopathic inflammatory myopathy characterized by severe muscle damage and minimal extra-muscular involvement. This report presents the first documented case of severe, treatment-resistant HMGCR-myopathy in a Tunisian and North African patient. A 43-year-old man with no significant medical history experienced progressive muscle weakness over one year, leading to difficulty walking. Examination revealed pronounced proximal muscle weakness, particularly in the lower limbs, with significant quadriceps atrophy. Laboratory results indicated elevated Creatine Kinase (CK) levels at 10000 UI/l and Lactate dehydrogenase (LDH) at 400 UI/l. Electromyography confirmed myogenic damage, and muscle biopsy revealed extensive muscle necrosis and regeneration with moderate inflammatory infiltrates. Screening for anti-HMGCR antibodies was positive. Initial treatment with high-dose prednisone showed a good response but led to flares upon tapering. Subsequent treatment with methotrexate, azathioprine, and rituximab resulted in partial clinical and biological improvement. This case underscores the challenges in diagnosing and managing anti-HMGCR myopathy due to limited awareness and access to testing.

## 1 Introduction

Idiopathic inflammatory myopathies are a rare type of connective tissue disease (CTD). They form a heterogeneous group with different clinical presentations and prognosis. A new categorization into 4 clusters has been suggested (1) which are dermatomyositis, anti-synthetase syndrome, inclusion bodies myositis and Immune-Mediated Necrotizing Myopathies (IMNM). IMNM has also been divided into 3 clinico-immunological phenotypes: anti-Signal Recognition Particle (anti-SRP) myopathy, anti-3-hydroxy-3-methylglutaryl-coenzyme A reductase (anti-HMGCR) myopathy and seronegative IMNM. On an international level, data regarding clinical associations, treatment outcome and guidelines anti-HMGCR IMNM are scarce considering their low prevalence estimated around 1.94-10.3 per million adults per year (2) and around 12% of inflammatory myopathies excluding Inclusion Body Myositis (IBM) (3). In Tunisia, diagnosis of this entity remains challenging and data are inexistent in this regard. Herein, we report a case of a severe and resistant HMCGR-myopathy in a Tunisian 43-year-old man.

## 2 Case description

A 43-year-old man with no medical history and no previous or current medication, presented with a one year history of progressive muscle weakness with difficulty of walking and standing without dysphagia or swallowing problems. Physical examination revealed the patient to be apyretic and eupneic. Cardio-pulmonary auscultation was normal. No skin lesions were found. The patient was fully oriented to time and space. Gait assessment revealed a waddling pattern, accompanied by marked atrophy of the quadriceps muscles. A proximal motor deficit was observed, predominantly involving the pelvic girdle, with positive Mingazzini and Barré signs. The patient was able to walk on heels and toes. Arm elevation and resistance to downward pressure were possible, although mild weakness was noted. Grip strength was preserved bilaterally. Osteotendinous reflexes were present and symmetrical in both upper and lower extremities. Deep and superficial sensory modalities were intact. Cranial nerve examination was unremarkable, and the swallowing test yielded negative results. The osteo-tendinous reflexes were present and symmetrical. No sensory deficit was observed. Muscle testing confirmed proximo-distal muscle weakness at both belts rated at 3. Chest X-ray was normal. Electrocardiogram did not reveal any abnormalities.

**Figure 1:**
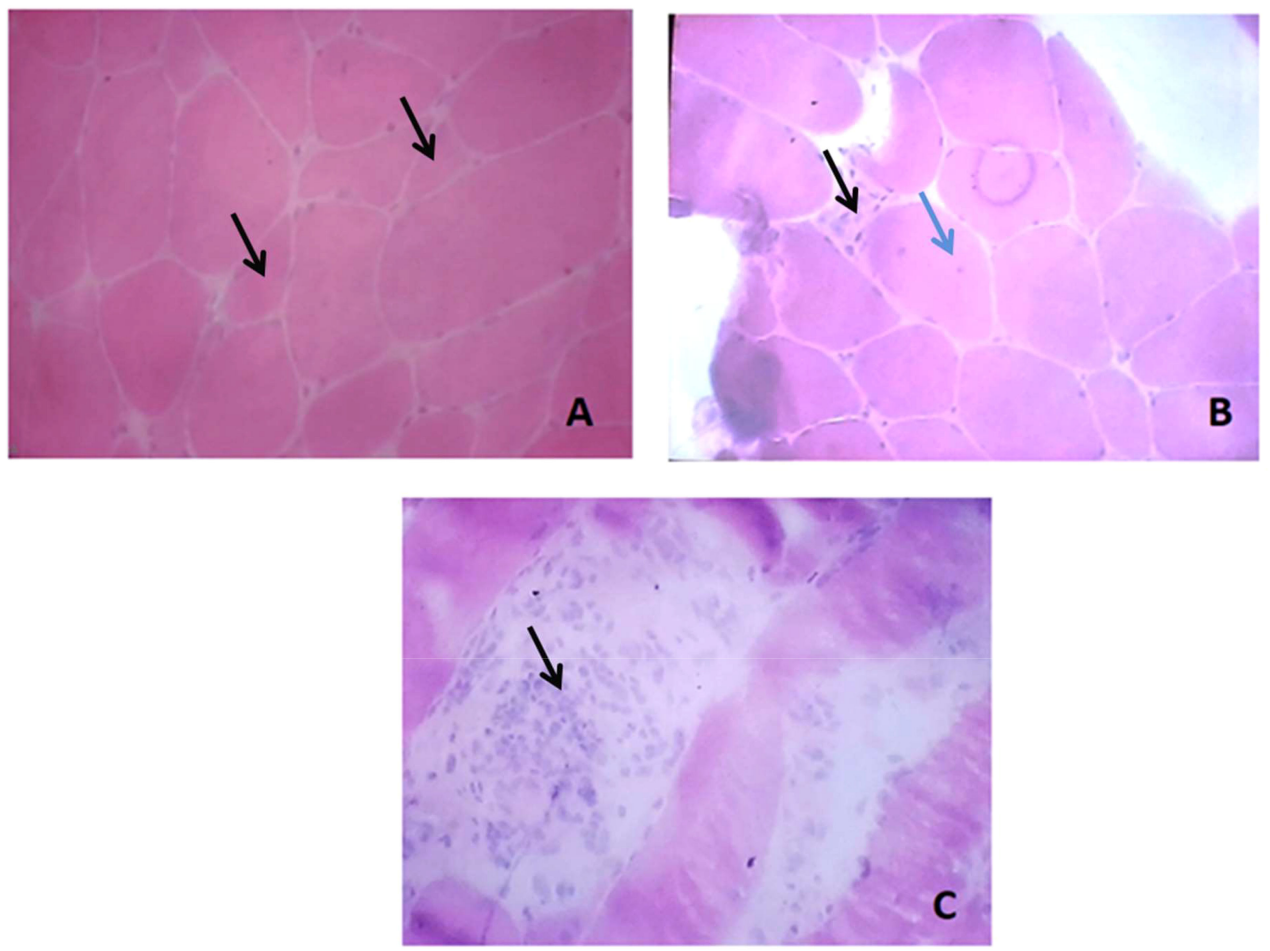
Muscle biopsy lesions: **(A)** Variation in fiber size with angulated atrophic fibers (black arrows); **(B)** Fibers with abnormal internalized nuclei (blue arrow) and another necrotic fiber (black arrow); **(C)** Phagocyte invading necrotic fibers with inflammatory cells infiltrates (black arrow).

The biological assessment revealed myolysis with Creatine Kinase (CK) at 10000 UI/l. Biological data are detailed in **Table 1**. Serological blood tests for Human Immunodeficiency Virus (HIV), Cytomegalovirus (CMV), Epstein-Barr Virus (EBV), Parvovirus B19, hepatitis B and C and lyme disease were negative. Antinuclear antibodies (ANA) screening using indirect immunofluorescence on HEp-2 cells was positive at a titer of 160 with a speckled pattern. Immunoblot assay to test myositis specific autoantibodies (MSAs) using Immunoline blot EUROLINE autoimmune inflammatory myopathies 16 Ag (IgG) profile (EUROIMMUN^®^, Lübeck, Germany) was negative. Anti-neutrophil cytoplasmic antibodies (ANCA) were negative. Electromyography (EMG) revealed myogenic damage in four limbs. Muscle biopsy in the left quadriceps muscle was conducted. Hematoxylin and eosin (H&E) and modified Gomoritrichrome stain section revealed unequal muscle fibers in size, with multiple diffuse muscle necrosis and regeneration as shown in **Figure 1**. Moderate intra-parenchymal inflammatory infiltrates were also found. Myosin adenosine triphosphatase (ATPase) histochemical staining showed Type II fibers were mostly predominant. Nicotinamide adenine dinucleotide tetrazoliumreductase (NADH) staining did not show any mitochondrial disorder. Immunohistochemical analysis for MHC-I on muscle biopsy was unavailable. Additional screening for anti-HMGCR by chemiluminescence (Werfen^®^) antibodies was positive at 272.9 UC (< 20 UC). Anti-HMGCR immune mediated necrotizing myopathy was thus retained.

**Table 1:** Patient’s biological data on disease onset.

Biological parameters	Result (reference range)
Creatine Kinase (CK) (IU/L)	10000 (55-170)
Lactate dehydrogenase (LDH) (IU/L)	400 (140-280)
Alanine transaminase (ALT) (IU/L)	80 (4-36)
Aspartate transaminase (AST) (IU/L)	70 (8-35)
Serum creatinine (µmol/l)	65 (65-120)
Serum potassium (mmol/l)	4 (3.5-4.5)
C-Reactive Protein (CRP) (mg/L)	6 (< 7)
Troponin (ng/mL)	4 (<19)
Thyroid-stimulating hormone (TSH) (mIU/L)	0.7 (0.4 - 4)
Sedimentation rate (mm/hour)	10 (<20)
Hemoglobin (g/dl)	13 (12-16)
Leucocytes (/mm)	4200 (4000-10 000)
Platelets (3×10^11^/unit)	160000 (150 000-450 000)

The patient was started on prednisone (1 mg/kg per day; 60 mg/day) and the response was initially positive as CK levels decreased to 1000 UI/l after 2 weeks of treatment with worsening of muscle weakness. Yet, they increased again when prednisone was tapered to 40 mg/day. Prednisone was maintained at the same dose of 40 mg/day and methotrexate was started at a dose of 15 mg/week. The patient did not show any marked clinical improvement and biological assessment showed an increase of CK levels to 14000 UI/l. Prednisone dose was thus increased to 60 mg/day and Intravenous immunoglobulins (IVIg) (2 mg/kg) and azathioprine (150 mg/day) were started. Methotrexate was discontinued after 3 months. CK levels showed marked decrease to 2000 UI/l after one month and prednisone tapering started progressively.

At three months follow-up, and with a dose of prednisone of 30 mg/day, the patient presented a flare with rapid worsening of muscle weakness and increase of CK to 2700 UI/l. The patient was screened for malignancies as part of the diagnostic work-up. Chest, abdominal, and pelvic CT scans were performed and showed diffuse muscle atrophy but no suspicious masses or lymphadenopathy suggestive of any underlying malignancy however, PET-CT imaging studies were not conducted. Body CT showed diffuse muscle atrophy but no suspicious masses or lymphadenopathy. After ruling out infectious triggers, Methylprednisolone infusions were started for three days followed by prednisone (1 mg/kg/day; 60 mg/day). Rituximab (RTX) was started (1 g on day 1 and 1 g on day 15). Azathioprine was maintained. At the follow-up one month after, CK levels showed partial improvement at 1700 UI/l. Methotrexate was resumed with progressive dose increase up to 25 mg/week. RTX was continued with a maintenance regimen (1 g every 4 months). Azathioprine was also maintained with blood concentration within therapeutic range. Slowly progressive tapering of prednisone was conducted. CK levels showed marked improvement. At the assessment before the 4th RTX infusion with a prednisone dose at 30 mg/day, the patient reported mild myalgia. Muscle testing showed stabilization of the weakness. CK titers were at 2670 UI/l and LDH 516UI/l. Prednisone tapering resumed. IVIg were suggested but were not accessible due to the patient’s limited medical health insurance.

From a patient perspective, the disease had a considerable psychological impact due to the professional and functional impairment.

## 3 Discussion

We report a case of a severe HMGCR-myopathy with diffuse muscle atrophy in a Tunisian 43-year-old man. This case represented a therapeutic challenge due to steroid dependence on one hand and resistance to immunosuppressive therapies on the other. Combined therapy with rituximab (RTX), methotrexate, and azathioprine managed to achieve partial improvement. To our knowledge, this case represents the first anti-HMGCR IMNM case to be reported in North Africa. This case underscores the underrepresentation of North African populations in current literature on idiopathic inflammatory myopathies, with limited epidemiological data from these reEnglish checkinggions contributing to diagnostic delays and underdiagnosis. The incidence of this condition in Caucasian populations ranges from 1.94 to 10.3 cases per million adults annually, depending on the immunoassays used (2).The lack of such data from African countries further exacerbates the challenge, hindering an understanding of ethnic influences on disease prevalence. Comparative analysis with similarly resource-limited settings indicates a potential gap in awareness and access to appropriate diagnostic tools. This data gap also impedes efforts to advocate for the inclusion of commercial detection immunoassays in public health budgets. Strengthening surveillance and reporting in these populations could provide more comprehensive insights into the global burden of anti-HMGCR myopathy and improve diagnostic and treatment strategies. Anti-HMGCR IMNM is considered to be among the least frequent subtypes of idiopathic inflammatory myopathies, estimated to represent around 12% after excluding inclusion body myositis (IBM) (3).The identification of anti-HMGCR antibodies dates back to 2010, when Christopher-Stine et al. reported a unique autoantibody specificity targeting 200 and 100 kDa proteins in patients exhibiting clinical and biological features of myositis (4). In fact, the HMGCR antigen is an oxidoreductase enzyme involved in the mevalonate pathway, specifically in the rate-limiting step in cholesterol synthesis. It is located at the membrane of the endoplasmic reticulum and is usually overexpressed in cancer tissues and regenerating muscle cells (5). On human epithelial type-2 (HEp-2) cells immunofluorescence, anti-HMGCR antibodies usually display a finely granular cytoplasmic appearance with perinuclear reinforcement. This aspect is observed in only 35% of patients (6). On kidney/stomach/liver substrates, these antibodies can have a scattered hepatocyte pattern with a centrolobular distribution. The staining is confined to the cytoplasm and clearly spares the nuclei. The bile ducts, endothelium, and sinusoidal cells are not stained. Stomach and kidney do not show any characteristic pattern (5). Initially, anti-HMGCR myopathy was thought to be statin-induced (4), but studies have shown this is unlikely, as only 15–44% of anti-HMGCR patients have a history of statin use (7). Statin-naive patients, often younger, are becoming more common, as with our patient. Anti-HMGCR IMNM mostly affects adults, though juvenile cases are reported, with rare overlap syndromes (8). Clinically, it primarily involves proximal muscle myalgia and weakness, sometimes affecting oropharyngeal muscles, leading to dysphagia and risk of aspiration pneumonia. Elevated CK levels, ranging from mild to severe, reflect myolysis and are useful for tracking treatment response and flares. In our patient, relapse was monitored using a combination of rising CK levels and clinical deterioration, including worsening proximal muscle weakness and functional decline (9). EMG usually reveals myogenic patterns in the proximal muscles. Imaging with muscle magnetic resonance imaging (MRI) is not always required for diagnosis but can serve multiple roles. First, it allows for the demonstration of edema reflecting muscle inflammation, usually localized to all compartments of the thighs with particular involvement of the anterior compartment. It can be more diffuse and asymmetrical. Edema also usually spares subcutaneous tissue and fascias, unlike in dermatomyositis (10). In addition to aiding in diagnosis, MRI assists surgeons in identifying accessible muscles with active lesions for biopsy when necessary. Furthermore, MRI can evaluate irreversible muscle damage, characterized by muscle atrophy with replacement by fat and connective tissue, as demonstrated in our patient’s body computed tomography (11). Similar to anti-SRP myopathy, it was initially suggested that anti-HMGCR titers correlate with muscle strength and CK level when studying all patients combined (12, 13). Yet, when analyzing sub-groups, the correlation between antibody titers and CK levels was not found in the statin-naive and statin-exposed groups in the Allenbach study (12). The correlation was found between titers and muscle strength only in statin-naive patients. In the Mammen study (4), correlations were found between antibody titers, muscle strength, and CK titers in statin-exposed patients. Finally, Aggarwal et al. have shown that antibody titers decrease with immunosuppressive treatment and might represent a follow-up marker. Yet, they seem to remain abnormal in patients even after clinical and biological remission (12). This study, along with similar findings, suggests a possible pathogenic role of anti-HMGCR in the pathophysiology of this myopathy. Larger studies are required to better assess this potential link. In Tunisia, screening immunoassays for this antibody remain unavailable due to limited public laboratory funding. This contributes not only to the underdiagnosis of the condition but also to the inadequate monitoring of disease activity, as illustrated in our case. Muscle biopsy is not systematically conducted. It can be indicated for diagnostic purposes, especially when anti-HMGCR antibody testing is not accessible. It typically shows a dystrophic pattern with necrosis/regeneration, associated with irregular fiber size and endomysial fibrosis—hallmarks of all IMNM. Immunohistochemistry may show class I major histocompatibility complex (MHC I) upregulation and deposition of the membrane attack complex (MAC) on the sarcolemma of non-necrotic muscle fibers. Interestingly, this MAC deposition was not found in anti-Signal Recognition Particle (SRP) IMNM biopsies and, as such, may be a characteristic feature of anti-HMGCR IMNM (8). In resource-limited settings, access to diagnostic tools such as antibody testing, muscle MRI, or even muscle biopsy is often limited by cost or availability. Treatment options may also be constrained, particularly for advanced therapies like IVIg or biologics. These barriers may delay diagnosis and optimal management. Developing standardized clinical criteria and treatment algorithms tailored to such settings could help bridge these gaps. Extra-muscular involvement in anti-HMGCR myopathy, like anti-SRP myopathy, is rare, as seen in our patient with a purely muscular presentation (8). Cardiac involvement, though frequent, is usually asymptomatic and mild. Allenbach’s study found conduction abnormalities in 12.9% of patients, with only one case of heart failure, diagnosed 20 years before anti-HMGCR positivity (7). ECG abnormalities were noted in 60% of those systematically tested (13). Dysphagia occurs in 20–50% of cases (14). Ocular involvement is also rare, with only two cases reported in 2024 (15). **Table 2** summarizes organ involvement in major cohorts. Patients with anti-HMGCR IMNM face a higher cancer risk than those with anti-SRP IMNM, but lower than seronegative cases (16). No guidelines exist for managing anti-HMGCR IMNM. Statin-induced cases may improve after discontinuation, but most require long-term treatment due to severity and frequent relapses. High-dose glucocorticoids, like prednisone or methylprednisolone pulses, remain the mainstay of treatment, though response varies, with some success using prednisone alone (12). Some experts recommend IVIg as first-line therapy, especially in refractory cases during prednisone taper, as seen in our patient. A report noted that IVIg monotherapy (without glucocorticoids) in three patients led to partial or full muscle strength recovery (4). Treppo’s trial in 16 patients showed that IVIg combined with glucocorticoids and/or methotrexate improved clinical and biological outcomes in over two-thirds within six months, with relapses mostly after treatment cessation (17). In our case, IVIg was used during the acute phase after treatment failure, showing an initial response, though long-term outcomes in a maintenance scheme are unknown. Azathioprine and methotrexate (3 mg/kg/day) are also recommended as first-line DMARDs, with good safety but uncertain efficacy. Recently, numerous case reports and observational studies have documented the use of rituximab in refractory cases, either as a third-line treatment or as a first-line option for severe presentations (18). However, its efficacy is still unclear, with results varying significantly across studies. Many studies have reported treatment failure, particularly for remission induction. In our case, the efficacy of rituximab cannot be definitively assessed, as it did not lead to improvement as part of the remission induction regimen. In our case, access to Rituximab and IVIg was limited by public health insurance policies, which authorize these therapies only for severe cases that have proven refractory to standard treatments such as methotrexate and azathioprine. Our case presents several limitations. First, patient adherence to treatment was not evaluated, nor were quality-of-life parameters or reported outcomes. Second, follow-up is short after the fourth rituximab infusion, not allowing full assessment of response. Finally, IVIg impact in a remission maintenance regimen was planned but not started because of limited access. This constraint underscores significant challenges in healthcare accessibility and suggests that classifying the condition as refractory may be premature. The patient demonstrated an initial positive response to IVIG, and the inability to continue this therapy due to financial limitations may have impacted the overall outcome.

**Table 2:** Prevalence of the different organs involvement in different cohorts in patients presenting with anti HMGCR myopathy.

Study (Year, Location)	Patients (n)	Mean Age (years)	Sex Ratio (M/W)	Statin Exposure (n, %)	Antibodies CK Correlations	Muscle Involvement (n, %)	Muscle Atrophy	Muscle Biopsy Findings	Dysphagia (n, %)	Cardiac Involvement (n, %)	Skin Involvement (n, %)	Lung Involvement (n, %)	Articular Involvement (n, %)	Inflammatory Markers	Cancer (n, %)	Treatment	Outcome
Mammen et al. (4) (2011, USA)	45	52 ± 16	0.73	30 (66.7%)	NA	43 (95.6%)	NA	Necrotizing myopathy in 100%; inflammation in 20%	NA	NA	NA	2 ILD (1 anti-JO-1)	NA	NA	NA	NA	NA
Werner et al. (19) (2012, USA)	55	NA	0.375	NA	Significant correlation with CK levels and strength	55 (100%)	NA	Necrotizing myopathy with minimal lymphocytic infiltration (38/53)	NA	NA	NA	NA	NA	NA	NA	Prednisone ± immuno-suppressants	No clinical improvement; CK decreased
Allenbach et al. (7) (2014, France)	45	48.9 ± 21.9	0.36	20 (44.4%)	Significant correlation with CK levels and strength	44 (97%)	10 (22.2%)	Necrotizing myopathy (42/97.6%)	12 (26.7%)	4 (conduction abnormalities)	Raynaud's (7/45)	1 (idiopathic fibrosis)	5 (11%)	Elevated CRP in 21%	5 (11%)	Prednisone (43), methotrexate (15), azathioprine (10), IVIg (17), rituximab (9), others	Frequent flares; 1 death
Watanabe et al. (8) (2016, Japan)	45	56.4 ± 18.8	0.45	8 (18%)	NA	45 (100%)	20 (44%)	Necrotic and regenerative fibers; minimal inflammation	20 (44%)	NA	2 (4%)	3 (7%)	NA	NA	NA	Prednisone, various immunosuppressants	NA
Aggarwal et al. (12) (2019, USA)	23	64.6 (55.1–73.4)	0.39	18 (78%)	Baseline titers correlated with CK levels, not strength	21 (90%)	NA	Necrotizing myopathy observed microscopically	NA	NA	NA	NA	NA	NA	2 (9%)	Prednisone ± methotrexate, IVIg (5)	73% full response
Szczesny et al. (14) (2022, Sweden)	13	52 (34–75)	11 (92%)	NA	NA	100%	NA	Necrotic fibers, regeneration, sparse inflammation	4 (31%)	NA	Gottron’s (6/13), heliotrope rash (2)	NA	Raynaud’s phenomenon (7/13)	2 (15%)	1 (8%)	Prednisone, azathioprine (8), IVIg (5)	All improved
Oh et al. (20) (2023, South Korea)	17	NA	0.3	10 (59%)	Significant association with CK levels	100%	NA	Necrotic and regenerative fibers; minimal inflammation (2 patients)	3 (18%)	2 (12%)	NA	NA	NA	NA	1 (6%)	Prednisone (15), methotrexate (7), IVIg (5)	Antibody titers decreased post-treatment

M/W, Men/Women; NA, Not available; CK, Creatine Kinase; ILD, Interstitial Lung Disease; CRP, C-Reactive protein; IVIg, Intravenous Immunoglobulin.

## 4 Conclusion

We report the first documentation of an anti-HMGCR IMNM in the North African population. Such reports are crucial to assess the differences in presentation and evolution of this myopathy sub-type in this ethnic group that is still underrepresented in international studies. Anti-HMGCR myopathy is often misdiagnosed due to several factors, primarily a lack of awareness and limited access to immunological testing. The delay in diagnosing necrotizing myopathy leads to a late initiation of immunosuppressive treatment and the development of severe, largely irreversible muscle damage. Also this case’s management was challenging and we exposed multiple pharmacological strategies. Literature shows divergent responses to various immunosuppressive therapies. Additionally, many studies do not represent diverse populations, which can lead to interpretation biases. Therefore, larger studies that include a wider range of populations are necessary to enhance our understanding of the disease and establish appropriate management guidelines.

## Data Availability

The original contributions presented in the study are included in the article/supplementary material. Further inquiries can be directed to the corresponding author.
